# Treatment and Healing of Leishmaniasis in a Wolf in Semi-Captivity Regime from an Educational Center of Zamora Province (Spain)

**DOI:** 10.3390/ani14101436

**Published:** 2024-05-11

**Authors:** Javier Merino-Goyenechea, Jesús Palacios-Alberti, Tomás Yanes-Martínez, María Martínez-Valladares, Rafael Balaña-Fouce

**Affiliations:** 1 Departamento de Ciencias Biomédicas, Instituto de Biomedicina (IBIOMED), Facultad de Veterinaria, Universidad de León, Campus de Vegazana s/n., 24071 León, Spain; rbalf@unileon.es; 2 Centro del Lobo Ibérico “Félix Rodríguez de la Fuente” Robledo de Sanabria, Puebla de Sanabria, 49393 Zamora, Spain; jesus.palacios@jcyl.es (J.P.-A.); tomasyanes@hotmail.com (T.Y.-M.); 3 Instituto de Ganadería de Montaña (CSIC-Universidad de León), Finca Marzanas, Ctra. León-Grulleros s/n., Grulleros, 24346 León, Spain; mmarva@unileon.es

**Keywords:** canine leishmaniasis, *Leishmania infantum*, wild animals, wolves, semi-captivity

## Abstract

**Simple Summary:**

*Leishmania infantum* is a single-celled trypanosomatid responsible for canine leishmaniasis, a serious disease that causes a generalized inflammatory reaction including, among others, lymphadenopathy, hepatomegaly, and splenomegaly in dogs. Increasing information on leishmaniasis in wildlife shows that many mammalian species can act as reservoirs, being a serious threat to domestic dog populations and public health. Our case describes a female wolf suffering from lameness due to an ulcerative wound on the right forepaw, with hyper-gammaglobulinemia, high serum GPT levels, and positive ELISA tests for antibodies to *Leishmania infantum* antigens as confirmed by PCR. The wolf responded positively to conventional treatment of leishmaniasis, and all altered parameters were restored.

**Abstract:**

Leishmaniasis in wild canids is a vector-borne disease caused in Europe by the protozoan parasite *Leishmania infantum*. To date, there is limited information on clinical signs and laboratory abnormalities in wolves due to leishmaniasis. The current clinical case report described a female Iberian wolf (*Canis lupus signatus*) housed in semi-captivity conditions at the Centro del Lobo Ibérico “Félix Rodríguez de la Fuente”, in Robledo de Sanabria, Zamora (Spain), with an interdigital ulcerous wound at the right forepaw, hyper-gammaglobulinemia, and abnormal liver blood parameters. Definitive serodiagnosis of leishmaniasis was established using antileishmanial serum antibodies and PCR analysis of different biological samples. A gold-standard anti-*L. infantum* treatment protocol consisting in subcutaneous meglumine antimoniate and oral allopurinol combination was installed. However, the presence of pain at the site of injection due to meglumine antimoniate administration forced its substitution by oral miltefosine. A progressive reduction of the levels of anti-*L. infantum* serum antibodies and the concentrations of gamma-globulin fraction was detected after antileishmanial treatment as well as a decline of liver GPT. To our knowledge, this is the first case of leishmaniasis diagnosed in a wolf housed in semi-captivity conditions, with the condition subsequently treated and successfully cured.

## 1. Introduction

Canine leishmaniasis caused by the protist *Leishmania infantum* (CanL) is a metazoonosis transmitted by phlebotomine sandfly vectors, endemic in the regions surrounding the Mediterranean basin [1,2,3]. In domestic dogs, CanL is a serious disorder that can be lethal if not adequately treated. Lesions are associated with a generalized inflammatory reaction and include peripheral lymphadenopathy and hepatosplenomegaly. In addition, dermatological signs include alopecia, dermatitis, onychogryphosis, and epistaxis. Renal disease is severe and may progress from nephrotic syndrome to chronic renal failure [4,5,6,7,8].

In addition to domestic dogs, numerous cases of wild mammals carrying *Leishmania* antibodies have been reported, including carnivores of different genera, bats, rodents, and lagomorphs, among others [9,10,11,12,13,14,15]. The importance of CanL should not only be addressed from the point of view of animal welfare but as a real public health problem since humans are also a definitive host of *Leishmania* and can undergo the potentially fatal visceral form of the disease. The relevance of this fact is reflected in the outbreak detected in Madrid (Spain) during the period 2009 to 2011, in which hares and rabbits were reported as necessary reservoirs of leishmaniasis cases suffered by humans, with an estimated incidence of 55.7 cases/100,000 inhabitants [16,17]. Therefore, it is of the utmost importance to prevent and eventually to eradicate the disease in all possible scenarios where it may arise, which implies close collaboration between authorities and health professionals.

Due to their phylogenetic similarity with dogs and their coexistence in environments where vectors reproduce, it is to be expected that wolves are potential reservoirs of *Leishmania*. To this must be added their social habits and their proximity to rural areas where dogs still play important guarding, herding, or hunting roles. Since the first description of leishmaniasis caused by *L. infantum* (MON-1 strain) in wolves in Croatia in 2008 [18], studies on the existence of this disease have been sporadic, unsystematic, and in most cases performed on remains of animals killed in hunts or traffic accidents or poisoned. In these animal remains, typical clinical signs of CanL found in dogs have been described, such as chronic dermatitis, hair loss, scabs, skin erosions, ulcerations, cachexia, orchitis, lymphadenopathy, hepatomegaly, and splenomegaly. In previous studies carried out to determine the endemicity of CanL in wolves in northwestern Spain, high seropositivity values were obtained both in animal remains in the province of Asturias [19,20] and in animals under semi-captivity during the period 2018–2022 in the province of Zamora [21]. In the latter case, the incidence of CanL was around 50% using a PCR-based diagnostic system. This percentage reached up to 57% when adding the positives found in other wolves captured alive, sampled, and subsequently released.

To date, apart from wild animals exhibited in zoos and circuses, no case of CanL in wolves that has been diagnosed and treated in semi-captivity conditions has been reported [22]. Due to the exceptional facilities offered by the Iberian Wolf Environmental Education Center “Félix Rodríguez de la Fuente”, which houses three wolf packs under sanitary control by specialized personnel, vector-borne diseases can be detected in conditions close to that of nature, which allows for their treatment. The endemicity of the vectors transmitting leishmaniasis in the region [23,24] opens the possibility of the occurrence of cases whose seroprevalence has already been reported in wolves but so far have not been reported as clinical cases. In the present report, we present the case of a wolf diagnosed with canine leishmaniasis, which was treated in time to normalize its seroprevalence and heal the wounds related to the infection.

## 2. Case Report

The case report is about a female Iberian wolf (*Canis lupus signatus*) born on 8 June 2012 and known as Dakota, housed in semi-captivity conditions at the Iberian Wolf Center “Félix Rodríguez de la Fuente” (IWC), in Robledo de Sanabria, Zamora (Spain). The IWC is an environmental education organization with the aim of informing the public about the biology, conservation, relationships with society, tourism, ecology, etc., of the Iberian wolf. IWC is geographically located in the “Sierra de La Culebra” at the northwest of Castilla y León (Spain) 41°59′35″ N, 6°34′25″ W, at 965 m altitude, a natural area of community interest, https://centrodellobo.es/ (accessed on 7 May 2024). The natural environment of the wolf specimens living in the center is a pine forest (*Pinus sylvestris*) of 20 Ha in surface divided into six enclosures interspersed with heathland and some chestnut, birch, and ash trees. The difference in height between the lowest and the highest part of the enclosures is 50 m.

The IWC maintains a stable population of fourteen wolves housed in semi-captivity conditions, distributed in three established wolf packs. The wolves are housed in three different enclosures, interacting with each other as well as with members of the management team. Routine management of the specimens housed at the IWC includes daily inspection during the administration of the food ration, where any signs of disease, injury, or behavioral disturbances are reported to specialist veterinary staff. In addition, an annual examination of the specimens is carried out under sedation. In this case, a comprehensive protocol including blood sampling for routine analysis and systematic inspection of each animal is carried out. Finally, all specimens are subjected to a strict schedule of external parasite removal with one pipette of Frontline^®^ (Boehringer Ingelheim, Toulouse, France) (containing 6.7 mg/kg body weight fipronil and 6 mg/kg body weight (S)-metopreno), internal deworming with one tablet per 10 kg body weight of DrontaPLUS SABOR^®^ (KVP Pharma, Kiel, Germany) (containing 15 mg febantel, 14.4 mg pyrantel embonate, and 5 mg praziquantel per kg body weight), and a vaccination protocol (Table 1).

Since 2013, the annual protocol shown in Table 1 includes the administration of an anti-rabies vaccine (Rabisyva Vp-13^®^, SYVA, Leon, Spain) and the vaccine Canigen DHPPiL^®^ (Virbac, Carros, France) to prevent parvovirosis, hepatitis, parainfluenza, distemper, and leptospirosis. It is worth mentioning that there is a specific protocol for wolves in which the tetravalent vaccine is applied at two months of age and a new revaccination one month later. This protocol may be subject to variations depending on the physiological situation of the animals and the existence of specific health alarms or regulatory constraints. Due to the increasing number of CanL cases recorded by veterinary practitioners in dogs in the region (unpublished results), the protocol was modified to include vaccination against *Leishmania* in the IWC wolves with CaniLeish^®^ (Virbac, Carros, France), the only commercial vaccine available at that time. This vaccine was administered to all wolves housed at the IWC from 15 May 2015. Then, vaccine boosters were administered annually according to the manufacturer’s recommendation for dogs. Prior to vaccination, all wolves were sedated for blood sampling with medetomidine (40 µg/kg body weight; Dorbene Vet^®^, SYVA, Leon, Spain) and ketamine (5 mg/kg body weight; Anesketin^®^, Eurovet Animal Health B.V., Bladel, Netherland), both administered by intramuscular injection. No anti-leishmanial antibody titers were found in any wolf by an indirect commercial ELISA method (LSH Ab Test Kit SensPERT^®^, Vetall Laboratories, Tongill-ro, Republic of Korea). The CaniLeish^®^ vaccine (Virbac, Carros, France) has secreted and excreted proteins from *Leishmania infantum* (PSE) as the active ingredient and purified extract of *Quillaja saponaria* (QA-21) as the adjuvant. The CaniLeish^®^ vaccine (Virbac, Carros, France) does not interfere in the diagnosis of LCan rapid tests, and the vaccine antibodies are not detected with the use of quantitative tests (IFA, ELISA) [25].

**Figure 1:**
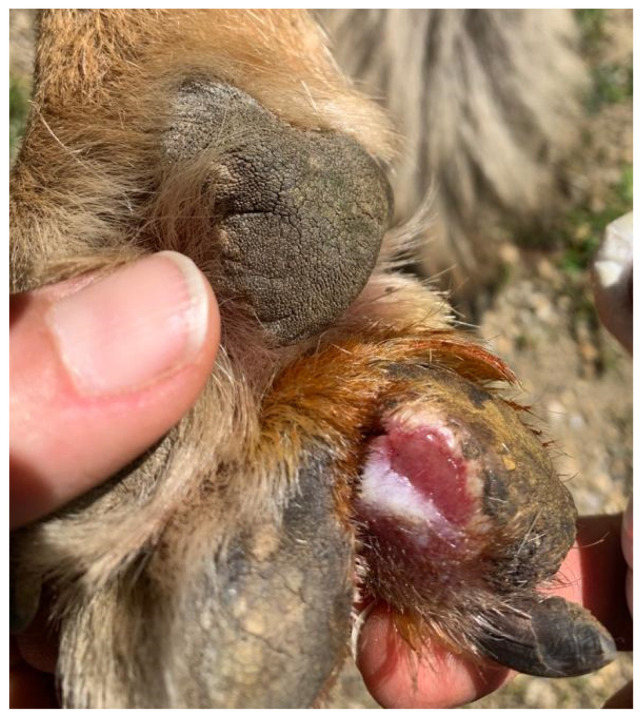
Visual examination of right forepaw of wolf Dakota showing interdigital ulcerous wound.

In February 2021, a case of lameness was observed in a 34.5 kg female wolf specimen that did not disappear with time. Since this animal was one of the most socialized wolves (meaning easy to handle by keepers without sedation), a quick examination of the paws was performed, and an interdigital ulcerative wound was detected on the right forepaw (Figure 1). Since the wound was detected during the pre-estrus period of the wolves, it was decided to isolate the animal and initiate a curative treatment based on daily washings, iodine cures, and a single subcutaneous injection of 8 mg/kg body weight of cevofecin (Convenia^®^) to prevent further infections.

### 2.1. Diagnosis and Initial Treatment of Leishmaniasis

On 23 March 2021, as no improvement was observed in the interdigital ulcerative wound after the treatment applied one month before, the specimen was subjected to a more exhaustive examination with the objective of obtaining an accurate diagnosis and establishing an appropriate treatment. For this purpose, the wolf was sedated following the protocol described above, and blood samples were collected for further analysis. No other notable clinical signs were observed at that time. Blood samples were collected by venoclysis from the cephalic vein: one tube with EDTA as anticoagulant for blood counts and another tube without anticoagulant for biochemical analysis and measurement of anti-Leishmania antibodies titers (Table 2). As the ulcerative wounds on the right forepaw were compatible with early skin lesions of CanL, in addition to the analysis by indirect ELISA, a rapid test for Leishmania diagnosis (Speed Leish ELISA kit K^®^, Virbac, Carros, France) was included in the analysis (Table 2). Speed Leish K^®^ detects circulating antibodies against *L. infantum* kinesins in blood, serum, or plasma samples. Blood samples were sent to a veterinary laboratory for clinical analysis (Laboratorio de Análisis Clínicos Sagunto, Valencia, Spain). Also, hair samples, an oral mucosal swab, and two swabs from both ears were also collected for in-house PCR analysis to amplify a 131 base pair fragment of the kinetoplast minicircle of L. infantum. The results of this PCR were shown in a previous study describing the prevalence of *L. infantum* in wolves sampled in Northwestern Spain [21].

The results of the indirect ELISA and the rapid test confirmed the presence of anti-Leishmania antibodies (Table 2). The antileishmanial circulating antibody response was initiated with a 1/640 titer by indirect ELISA (negative control >1/80 dilution, according to the manufacturer). Also, the analysis of buccal and ear swab samples by PCR showed amplification of the expected kDNA band specific for *L. infantum* [21]. However, the PCR in the hair sample resulted negative, showing the lower sensitivity of this PCR for this sample.

After analysis of biochemical parameters and serum protein electrophoresis, the main findings were hyper-gammaglobulinemia, at 18.3 g/L (range 3.3–10.6), and a significant increase in serum glutamic pyruvic transaminase (GPT), at 652 U/L (range 10–65), which could indicate liver damage. The other biochemical parameters were within reference values, showing that there was no possible renal damage from infection. On the other hand, blood counts were within reference values for dogs.

After cleaning and healing the paw wound, as described previously, one external pipette of Frontline^®^ was applied to prevent, as far as possible, reinfections of *L. infantum* transmitted by phlebotomine flies. In addition, antibiotherapy with marbofloxacin (2 mg/kg/day; Marbocyl^®^, Vetoquinol, Lure, France) was administered orally for 6 days to protect against opportunistic bacterial infections.

**Figure 2:**
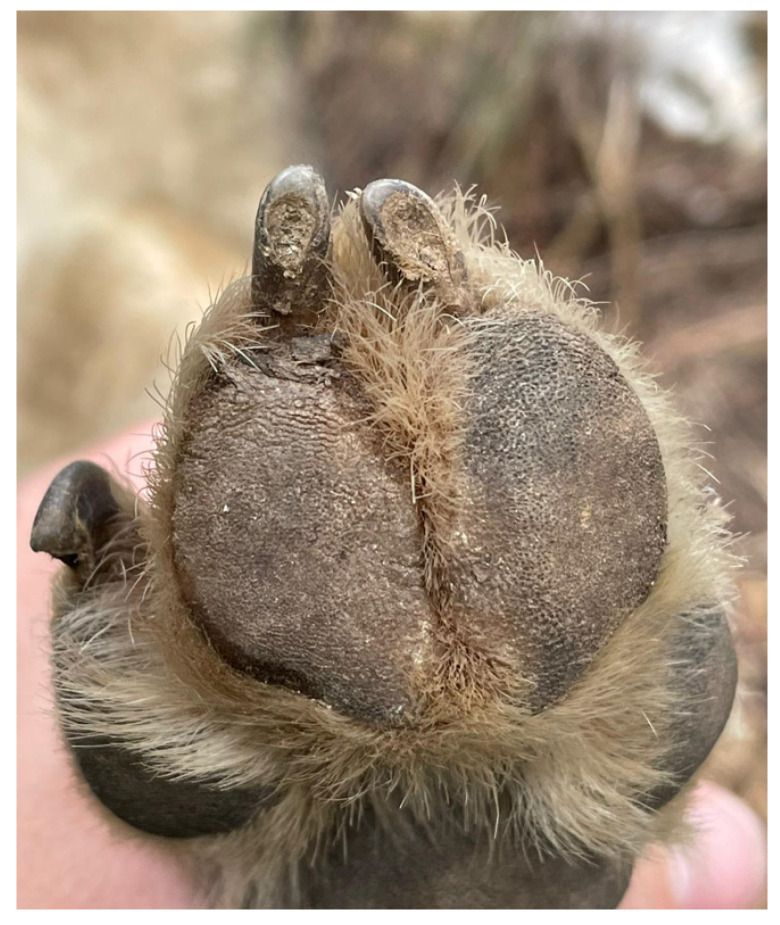
Visual examination of the right foreleg of the wolf Dakota showing healing of the interdigital ulcerative wound.

Immediately after confirming the diagnosis of CanL, a conventional antileishmanial treatment was applied by administering a combination of meglumine antimoniate and allopurinol (gold standard of CanL treatments in dogs) [3,26]. The doses administered were 10 mg/kg body weight/day of allopurinol (Zyloric^®^, Faes Farma, Leioa, Spain) (in two administrations every 12 h) orally during 12 weeks and 100 mg/kg body weight/day of meglumine antimoniate (Glucantime^®^, Sanofi-Aventis, Alcorcon, Spain) by subcutaneous route. Although the “guidelines” indicate that the treatment with meglumine antimoniate should be 30 days [26], in this case, we had to stop after 6 days due to the non-tolerance of the administration and the aggressiveness that the animal began to show, presumably due to pain at the application site. Therefore, it was decided to change the parenteral treatment for a more friendly oral treatment based on miltefosine (2 mg/kg/day; Milteforan^®^, Virbac, Carros, Spain) for 30 days. The oral administration of any drug was carried out with the help of “treats” (cheese or sausages). At the end of this protocol, the interdigital wound had evolved favorably and healed completely (Figure 2).

### 2.2. Evolution of the Clinical Case of Leishmaniasis 

On 24 September 2021, a second sampling was performed to determine blood biochemical parameters (Table 2). It was found that circulating anti-Leishmania antibodies remained elevated, with a 1/340 titer. Mild hyper-gammaglobulinemia at 9.8 g/L (range 3.3–10.6 g/L) but 15.6% (range 6.0–13.0%) and significantly elevated serum GPT values, 102 U/L (range 10–65 U/L) were also detected. According to these values, it was decided to continue the treatment with allopurinol until the end of 2021, with the same regimen established in March 2021. Therefore, from that moment, the administration of allopurinol lasted 36 weeks.

This trend towards improvement was verified in the third and fourth examinations performed on 6 May 2022 and 6 July 2023, respectively. The anti-Leishmania antibody titer decreased below positivity (PI > 1/80), although gammaglobulinemia and GPT values remained slightly higher than the reference values for the species.

## 3. Discussion

The progress of Dakota’s health status was optimal, responding to the established treatment. No clinical symptoms were observed after healing. Dakota’s follow-up shows the typical alterations in the biochemical and hematological analyses detected in CanL-seropositive dogs. Hyperproteinemia [27,28], due to a hyperglobulinemia that corresponds to the synthesis of antibodies against *Leishmania*, stands out. Hyper-gammaglobulinemia is a very characteristic alteration of CanL. An increase in the alpha-2-globulin fraction [28] and beta and gamma globulins [27], alteration of the albumin/globulin ratio [29], and a decrease in serum albumin (hypoalbuminemia) have been described in dogs [30]. In addition, monitoring serum electrophoretic values enables the evolution of the treated animal to be evaluated from the time of diagnosis and to assess how they progressively normalize because of the treatment administered. The albumin/globulin ratio was slightly lower than the reference values, which is consistent with a decrease in albumin concentration, possibly due to incipient damage to the renal glomerulus because of immunocomplex deposition. In severe cases, glomerulonephritis with albuminemia may lead to nephrotic syndrome with severe hyperproteinemia, which was not observed in our case.

Another aspect that can be found in blood tests of dogs with CanL is an elevated hepatic GPT [30]. Increased GPT values can be interpreted as a consequence of severe liver damage, as the GPT value in the blood is directly proportional to the amount of damaged tissue. In the second analysis, 6 months after treatment, GPT levels were still above baseline values, although they had decreased significantly compared to the first measurement (102/65). In the third analysis, it was observed that the GPT continued to decrease to 93 U/L, until normalizing below 65 U/L of baseline norms in the fourth sampling, indicating reversal of liver damage due to the treatment.

Ulcerative dermatitis can have several presentations. Erosive and ulcerative lesions may be observed at mucocutaneous junctions (all junctions may be affected). Ulcers have also been described in areas previously injured by self-induced or iatrogenic trauma [31,32]. Finally, ulcerative dermatitis may be secondary to cutaneous vasculitis because of immunocomplex deposition, in which case ulcers are located in distal areas of the body such as the tips of the ears, tail, toes, and pads [25].

As Dakota’s right forepaw wound and biochemical parameters were compatible with CanL, we performed an immunological test to detect antibodies against *Leishmania* and determine that the wolf was indeed infected. In the first test, positivity was established at a serum dilution of 1/640, which confirmed the reaction of the animal’s antibodies with the parasite antigens. Although the number of drugs to tackle leishmaniasis is not large [33], the meglumine antimoniate/alopurinol combination is considered the gold standard for CanL in dogs [3,26]. As described in the previous section, drug combination was initiated—with the meglumine antimoniate having to be replaced by miltefosine, as described below. By the second test, the anti-Leishmania antibody titer had dropped to 1/320, and by the third (1/160) and by the fourth (>1/80), levels considered negative by the kit manufacturer were reached.

It should be noted that, although the wolves at the IWC were vaccinated with CaniLeish^®^—the only vaccine available against CanL in 2015—several seropositive wolves had been observed in addition to the clinical case in Dakota. CaniLeish^®^ is an injectable formulation composed of excreted-secreted proteins from *L*. *infantum* (LiESP) supplemented with saponin-derived adjuvants such as QA-21. To our knowledge, CaniLeish^®^ had not been applied systematically in a wolf community under semi-captive conditions. In addition to promoting an increase in humoral IgG2 levels, CaniLeish^®^ induces a strong shift towards a Th1 immune response in dogs [34,35]. After primovaccination, blood levels of IG2 and IFN-γ persisted for approximately one full year, and this may be the reason why all recorded cases were mild [34]. Since 2022, the IWC vaccination protocol has used the Letifend^®^ vaccine (LETI Pharma, Tres Cantos, Spain), which has significant published results in terms of protection against CanL and is better tolerated by the animals [36,37].

When we initiated anti-leishmanial treatment in Dakota, immediately after serological diagnosis, we used the gold-standard dog combination of meglumine antimoniate and allopurinol, which we assumed would also work in wolves. However, the wolf developed aggressive behavior towards the caretaker in charge of injecting meglumine antimoniate subcutaneously (possibly because of pain at the injection site), which had to be replaced by miltefosine. Miltefosine, a second-choice drug, is the only drug that can be administered orally against *Leishmania*, either as monotherapy or in combination with other leishmaniostatic drugs, and has replaced antimony derivatives as the first-choice treatment for CanL in southern European countries [1]. Several clinical trials show that miltefosine as monotherapy or in combination has a good therapeutic profile [38], contributing to an improvement of most clinical symptoms in dogs up to two years after drug withdrawal [39].

The allopurinol administration was maintained for the entire duration of Dakota’s treatment. In dogs, allopurinol is administered orally in the treatment of CanL, either alone as monotherapy or in combination with other drugs [3]. Allopurinol has leishmaniostatic effects, reducing the parasite load and thus preventing future relapses [26], with little toxicity to the host. The duration of treatment is variable and is adjusted to the positive evolution of clinical signs. In general, good results are obtained after prolonged treatment (4–10 weeks) with the above regimen, but relapses usually occur 2–4 weeks after drug withdrawal [40,41]. In combination with other first-line drugs, namely meglumine antimoniate or miltefosine, seropositivity is persistently reduced, and signs of CanL can be effectively reversed without relapses.

## 4. Conclusions

In the current clinical report of leishmaniasis in a semi-captive housed female wolf, the only adverse observed effect found was the presence of an interdigital ulceration on the right forepaw. Blood tests showed hyper-gammaglobulinemia and increased blood GPT, but no signs of renal damage were observed. The diagnosis was obtained by an ELISA blood test for anti-*Leishmania* antibodies confirmed by a previously reported in-house PCR analysis of mouth and ears swabs. The animal responded rapidly to treatment with a meglumine antimoniate/alopurinol regimen. Meglumine antimoniate was given by subcutaneous route and had to be replaced after few administrations by the oral administration of miltefosine due to the animal’s aggressiveness caused by the pain associated at the site of the injection. In our case, a good clinical response to treatment was detected after starting the anti-*Leishmania* treatment, healing the wound and restoring all biochemical parameters to normal values.

At 6 months after completion of treatment, the animal was still seropositive (May 2022), and it was not until a year later (July 2023) that it was seronegative. Unfortunately, there are no intermediate analyses to determine the duration of antibodies in the blood after treatment and no PCR data to determine if the infection or the parasite was completely eliminated after treatment and when.

## Figures and Tables

**Table 1:** Annual health plan for wolf disease control at IWC.

Treatments	Annual Health Plan for Disease Control Per Month					
	January	February	March	April	May	June
External parasites			Frontline^®^	Frontline^®^	Frontline^®^	Frontline^®^
Internal parasites	DrontalP^®^ (KVP Pharma, Kiel, Germany)			DrontalP^®^ (KVP Pharma, Kiel, Germany)		
Vaccines		Canigen^®^ (Virbac, Carros, France)			Rabisyva Vp-13^®^ (SYVA, Leon, Spain) CaniLeish^®^ (Virbac, Carros, France)	

**Table 2:** Body weight, biochemical parameters, and serology determined in the leishmaniotic wolf at the first veterinary examination before treatment and during follow-up. Values exceeding the reference values are in bold. The end of treatment was 15 December 2021.

Parameters	Antileishmanial Treatment				Reference Values
	25 March 2021	28 September 2021	6 May 2022	6 July 2023	
Body weight (kg)	34.5	35.0	35.3	36.0	25.0–38.0
BIOCHEMISTRY					
Blood Urea Nitrogen (mg/dL)	50.0	48.0	42.0	19,8	20.0–65.0
Creatinine (mg/dL)	1.37	1.04	1.45	1.27	0.5–1.5
ENZYMES					
Glutamic pyruvic transaminase, GPT (U/L)	**652.0**	**102.0**	**93.0**	**87.0**	10.0–65.0
PROTEINOGRAM					
Total proteins (g/L)	78.0	63.0	61.0	59.8	55.0–82.0
Albumin (%)	40.9	46.5	46.3	46.8	40.0–60.0
Alpha 1 globulins (%)	4.2	4.4	4.7	5.3	1.0–7.0
Alpha 2 globulins (%)	12.9	12.3	10.7	8.1	4.0–15.0
Beta globulins (%)	18.6	21.2	19.9	21.2	8.0–28.0
Gamma globulin (%)	**23.4**	**15.6**	**16.8**	**18.6**	6.0–13.0
Albumin (g/L)	31.9	29.3	27.8	28.0	22.0–49.0
Alpha 1 globulins (g/L)	3.3	2.8	3.1	3.2	1.6–5.7
Alpha 2 globulins (g/L)	10.1	7.7	5.7	4.8	2.2–12.3
Beta globulins (g/L)	14.5	13.4	14.2	12.7	4.4–23.0
Gamma globulins (g/L)	**18.3**	9.8	7.9	**1.11**	3.3–10.6
A/G ratio	**0.69**	0.87	0.91	0.88	0.70–1.50
Anti-*Leishmania* antibodies					
Speed Leish ELISA Kit K^®^	1/640	1/320	1/160	<1/80	>1/80

## Data Availability

Data is contained within the article.
